# Disparities in maternal and fetal health among ethnogeographic groups—a retrospective cohort study of 30,000 women from a middle eastern multiethnic maternity population

**DOI:** 10.3389/fgwh.2026.1708103

**Published:** 2026-08-17

**Authors:** Fathima Minisha, Salwa Abu Yaqoub, Najat Khenyab, Husam Salama, Sawsan AlObaidly, Nader Aldewik, Hilal Al Rifai, Thomas Farrell

**Affiliations:** 1 Department of Obstetrics and Gynaecology, Women’s Wellness and Research Centre, Hamad Medical Corporation, Doha, Qatar; 2 Department of Research, Women’s Wellness and Research Centre, Hamad Medical Corporation, Doha, Qatar; 3 College of Medicine, Qatar University, Doha, Qatar; 4 Department of Pediatrics and Neonatology, Women’s Wellness and Research Centre, Hamad Medical Corporation, Doha, Qatar

**Keywords:** adverse perinatal and neonatal outcomes, ethnicity, gestational diabetes, nationality, preeclampsia, stillbirth, VBAC (vaginal birth after cesarean section)

## Abstract

**Objectives:**

Maternal ethnogeographic origins and ancestry can influence perinatal health. This study evaluated the impact of nationalities grouped into geographic categories on maternal and neonatal outcomes.

**Study design:**

The study employed a retrospective birth cohort design.

**Methods:**

A total of 30,667 pregnant women and their neonates born at more than 24 weeks’ gestational age in secondary and tertiary government maternity hospitals in Qatar were included. Data were extracted from a preexisting maternity registry. Women were classified into seven ethnogeographic groups based on internationally accepted classifications: Qatar group (QG), Gulf Cooperation Council (GCC), Arab Region (AR), South/Central Asia (SA), southeast Asian (SEA), African (AF), and European/American countries. Antenatal, intrapartum, and neonatal outcomes were compared between the groups using adjusted multivariable regression models with QG as the baseline.

**Results:**

Participants represented 124 countries, with the majority from AR (37.6%). After Qatar (23.7%), the most represented countries were Egypt (12.3%) and India (11.6%). Gestational diabetes was most common in SA at 33.4% [adjusted odds ratio (aOR)=1.56, confidence intervals (CI) = 1.44–1.68; *p* < 0.0001], whereas SEA had higher odds of preeclampsia (aOR=2.50, CI = 1.86–3.36; *p* < 0.0001) and postpartum hemorrhage (aOR=1.88, CI = 1.55–2.28; *p* < 0.0001). Postdated pregnancy was more frequent in AF (aOR=2.34, CI = 1.85–3.0; *p* < 0.0001), while preterm birth rates were similar across groups. Vaginal birth after cesarean was highest in GCC (42.1%) and lowest in SEA and Africa (19%). Neonatal intensive care admission among term babies was similar across groups. No clinically relevant differences between groups were observed for rare neonatal outcomes such as stillbirth, neonatal death, or anomalies.

**Conclusion:**

Clinically relevant differences in pregnancy outcomes exist among nationality groups, potentially attributable to ethnicity, genetic susceptibility, and potential socioeconomic factors. Understanding these differences is essential for adequate risk assessment, and further genetic and anthropological studies are warranted to confirm these findings.

## Introduction

The term “race” was originally designed to promote societal class divisions based on physical traits. Genetic studies have reported that humans are approximately 99.9% genetically similar, indicating that race has limited biological impact (1). However, differences in external features and inherent susceptibilities result from allele changes inherited through family lines and are most likely influenced by geographical, environmental, and cultural factors—commonly referred to as ethnicity, which is closely linked to nationality.

Many studies have explored the impact of race on pregnancy outcomes, highlighting inequities in socioeconomic status (SES) and timely access to medical care (2). For example, studies from the US report higher maternal mortality and infant mortality among mothers of African origin (3). Similarly, in the United Kingdom, disparities have been observed in the incidence of gestational diabetes (GDM), hypertension in pregnancy (HIP), preterm births (PTB), and stillbirths among the different nationalities, with deprivation among minorities being a risk factor (4). Most studies attribute these differences to social inequalities affecting access to medical care and poor prepregnancy health due to increased malnourishment and substance abuse. However, beyond these factors, women from different countries are exposed to different cultures, lifestyles and genetic susceptibilities (5), which could have an impact on pregnancy outcomes.

Qatar has a multicultural expatriate population, with women from over 100 countries delivering in secondary and tertiary hospitals annually. Maternity services are free and accessible to all residents, regardless of SES. Comparing pregnancy outcomes among women of different nationalities who deliver in the middle- to high-income society of Qatar provides great insight into the impact of nationality on maternal outcomes. This study aims to assess the differences in antenatal, intrapartum, and postpartum outcomes among women from different ethnogeographic areas delivering in Qatar, after adjusting for maternal demographic characteristics.

## Methods

### Study design and participants

A retrospective, population-based birth cohort study was performed, enrolling women delivering in the government hospitals in Qatar between 2017 and 2018. Approval was obtained from the Institutional Review Board of Hamad Medical Corporation (MRC-01-24-107), with a waiver of informed consent since only anonymized preexisting data was used for analysis. The strengthening the reporting of observational studies in epidemiology (STROBE) checklist and the guidelines for reporting ethnicity in medicine were followed to prepare this manuscript (5, 6).

**Figure 1:**
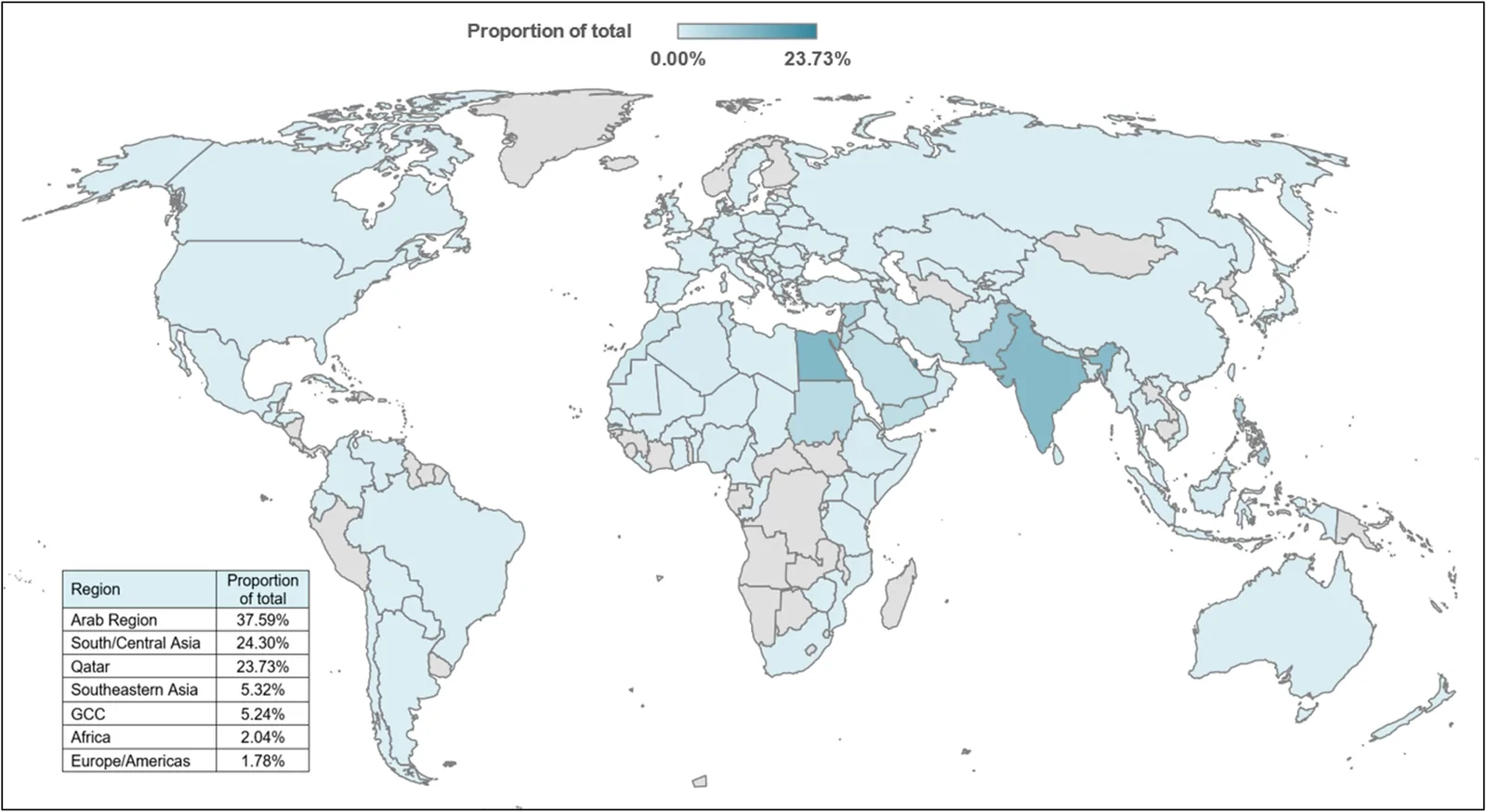
Depiction of the nationalities of women delivering in Qatar, as per proportions of the total (geographical areas in the table). *Arab Region*: Algeria, Djibouti, Egypt, Iraq, Jordan, Lebanon, Libya, Mauritania, Morocco, Palestine, Somalia, Sudan, Syria, Tunisia, and Yemen. *South/Central Asia*: Afghanistan, Bangladesh, India, Iran, Nepal, Pakistan, Sri Lanka, Uzbekistan, Kazakhstan, and Tajikistan. *Southeastern Asia*: the Philippines, Indonesia, Myanmar, Malaysia, Singapore, Thailand, China, Japan, Korea, and Taiwan. *GCC*: Bahrain, Kuwait, Oman, Saudi Arabia, and United Arab Emirates. *Africa*: Ethiopia, Eritrea, Kenya, Ghana, Nigeria, South Africa, Zimbabwe, Senegal, Chad, etc., (21 countries). *Europe/Americas*: United Kingdom, European Union including Turkey, Russia, USA, Canada, Mexico, countries of South America (56 countries).

Women with viable pregnancies (≥24 weeks’ gestational age, GA) were included in the study. The GA was determined by first-trimester dating ultrasound scans or the last menstrual period, if the former was unavailable. Those with missing data for exposures and outcomes and second pregnancies within the study period were excluded. Participants were divided into ethnogeographic groups based on citizenship documented in health records. The baseline group for all comparisons was the Qatar group (QG). Other nationalities were grouped based on the following internationally accepted geographic and cultural groupings, as also shown in Figure 1:

- Gulf Cooperation Council (GCC) countries: countries forming the Arabian Peninsula (7) Saudi Arabia, Bahrain, United Arab Emirates, Oman, and Kuwait.
- Arab Region (AR): countries of the Arab League (8), Algeria, Comoros, Djibouti, Egypt, Iraq, Jordan, Lebanon, Libya, Mauritania, Morocco, Palestine, Somalia, Sudan, Syria, Tunisia, and Yemen.
- South/Central Asia (SA) based on the United Nations (UN) geographic division (9): Afghanistan, Bangladesh, India, Iran, Nepal, Pakistan, Sri Lanka, Uzbekistan, Kazakhstan, and Tajikistan.
- Southeast Asia (SEA) based on UN geographic classification (9): the Philippines, Indonesia, Myanmar, Malaysia, Singapore, Thailand, Vietnam, China, Japan, Korea, and Taiwan.
- Africa (AF) including other countries of Sub-Saharan Africa: Ethiopia, Eritrea, Kenya, Ghana, Nigeria, South Africa, Zimbabwe, Senegal, Chad, etc. (21 countries).
- Europe/Americas (EA): United Kingdom, European Union including Turkey, Russia, North America, Mexico, Caribbean, Countries of South America, Australia, etc. (56 countries grouped together due to small numbers).

Data were extracted from a national maternity registry incorporating information from electronic patient health records. Maternal demographic variables included were maternal age, maternal body mass index (BMI) at the first antenatal visit and at delivery in kg/m^2^, parity (nulliparous: parity 0, multiparous: parity 1–4, grand multiparous: parity > 4), assisted reproduction techniques (ART) for conception, preexisting medical illness, and place of delivery (central tertiary or peripheral secondary care).

The antenatal outcomes explored were as follows: GDM: abnormal 75-g glucose tolerance test; HIP: including gestational hypertension and preeclampsia, defined as new-onset high blood pressure after 20 weeks’ GA with or without proteinuria and other organ involvement; cholestasis of pregnancy (COP): unexplained high liver enzymes and bile acids in the third trimester; and anemia: low hemoglobin during pregnancy. The delivery outcomes included incidence of cesarean section (CS); successful vaginal birth after cesarean (VBAC) in those with one prior cesarean; instrumental deliveries (forceps or vacuum) among those having vaginal births; GA at delivery in completed weeks; PTB if GA < 37 weeks; postdated births if GA > 40 weeks; and postpartum hemorrhage (PPH), defined as blood loss >500 mL after a vaginal birth and >1,000 mL after a cesarean.

Fetal growth outcomes included birthweight in grams; low birthweight (LBW) (birthweight <2,500 g in term babies); macrosomia (birthweight ≥4,000 g); and birthweight centiles customized according to maternal height, weight, parity, nationality, GA at birth, and biological sex of the baby (CBW). Small-for-gestation-age baby (SGA) was defined as CBW centiles <10% and large-for-gestation-age baby (LGA) as CBW centiles ≥90%. Neonatal outcomes included major congenital and chromosomal anomalies, stillbirths defined as any viable baby born with no signs of life, neonatal deaths defined as death within 28 days of birth in singleton pregnancies, and admission to neonatal intensive care unit (NICU) in term babies.

### Data analysis

Means and standard deviations (SD) were used to express continuous variables, which were compared using ANOVA with Bonferroni-adjusted *p*-values to account for multiple comparisons. Birthweight centiles were represented as medians and interquartile ranges (IQR), due to heavy-tailed distributions, and compared using the Kruskal–Wallis test. Frequencies and percentages were reported for categorical variables and compared using the Chi-square test (overall *p*-value reported comparing all exposure groups). Rare neonatal outcomes were reported per 1,000 births. Analyses of mode of delivery, growth, and neonatal outcomes were restricted to singleton births.

Binary outcomes were compared between exposure groups using logistic regression models, accounting for maternal demographics, to obtain adjusted odds ratios (aORs) with 95% confidence intervals (CIs). The variables adjusted for included maternal age, parity, BMI, preexisting comorbidities, location of antenatal care, ART, and previous cesarean (only for mode of delivery). Only variables considered potential confounders and known to be independently associated with the outcomes were included in the models. Pairwise correlations between variables were examined, and highly correlated variables were not included together in the models to avoid multicollinearity. For example, BMI, rather than maternal weight and height, was included in the model. Given the large sample size, with over 500 participants in the smallest exposure group, the study was well powered (80%) to detect clinically relevant differences for all outcomes at an alpha of 0.05.

Exposure groups had no missing data, as every individual residing in the country has a documented nationality. For other variables, the large sample size permitted us to perform complete-case analysis. Regardless, all the main outcome variables had <1% missing data. A two-tailed *p*-value <0.05 was considered strong evidence against a null hypothesis of no difference. Analyses were performed using Stata Release 18 (Stata Corp, Texas, USA) (10).

## Results

**Figure 2:**
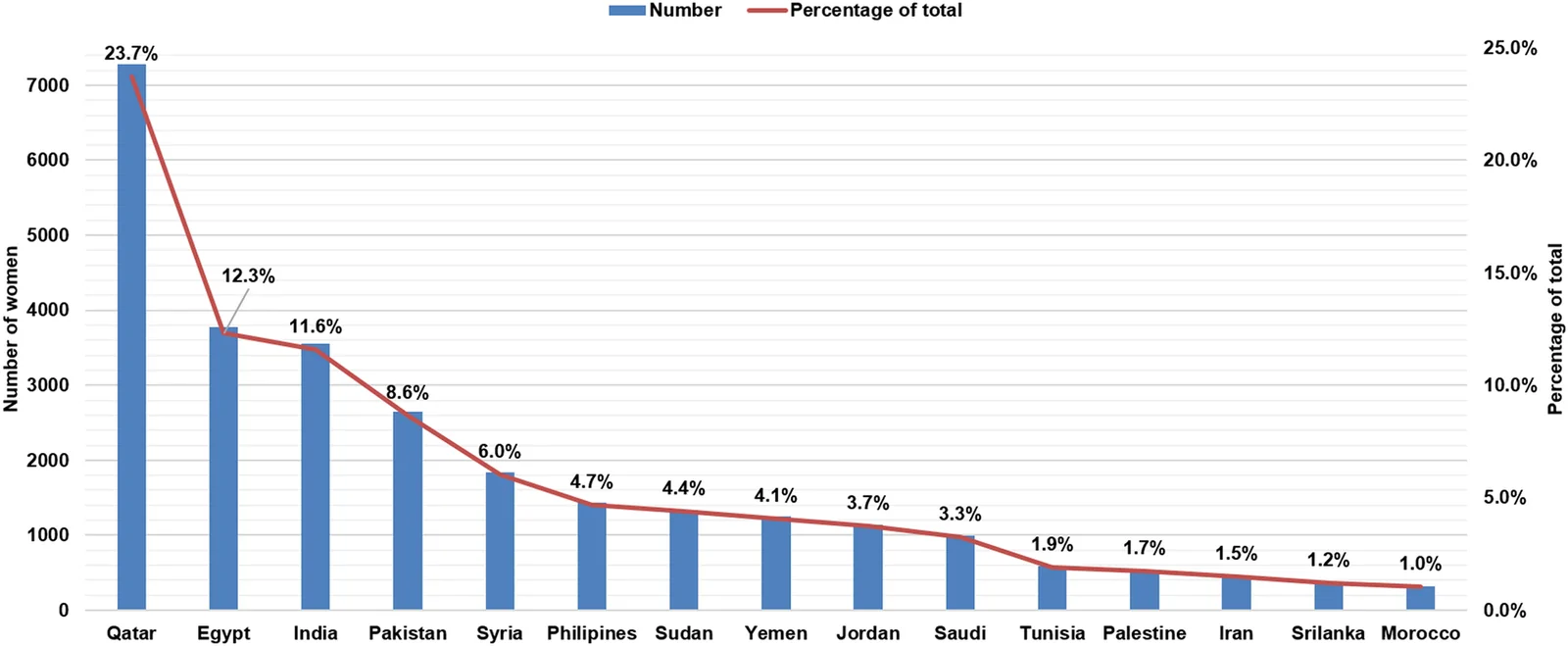
Nationalities of 90% of the women (*N* = 27,530) delivering in Qatar over 16 months, with percentages of the total in the cohort (total cohort *N* = 30,667).

A total of 30,667 women and their neonates were included in this study (Figure 1). The largest ethnogeographic area represented was AR (37.6%), followed by SA (24.3%). The highest proportions of women were Qatari (23.7%), followed by Egyptian (12.3%) and Indian (11.6%), with 90% of women belonging to 15 nationalities (Figure 2). The smallest groups were women from Africa (2.0%) and Europe/Americas (1.8%). The country leading the GCC states in representation was Saudi Arabia (3.3%), with Egypt and India leading the AR and SA groups, respectively. The country dominating SEA was the Philippines (4.7%), while Ethiopia (0.8%) and England (0.4%) led the AF and EA groups, respectively.

The mean maternal age was lowest in GCC and highest in SEA (Table 1), with more than 30% of SEA and EA women aged ≥35 years. Approximately 61.9% of women in QG belonged to the obese category (BMI≥30 kg/m^2^) at delivery, followed closely by AR and GCC, compared with 38.9% in SEA. Nulliparity was highest in AF, with 13.6% of QG being grand multiparous, nearly three times higher than GCC, AR, and SA. In multiparous women, nearly 40% of EA had a previous cesarean compared with only 25.9% in GCC. While preexisting diabetes was highest in QG, SEA had a fourfold higher prevalence of chronic hypertension compared with other groups. More than 60% of women had government-sector booking visits by the second trimester, the highest being 73.5% in QG.

**Table 1:** Maternal demographics in the nationality groups.

Maternal demographics		Qatar *N* = 7,277	GCC *N* = 1,608	Arab *N* = 11,527	South Asia *N* = 7,452	SE Asia *N* = 1,631	Africa *N* = 627	Eur/ Ame *N* = 545
Age in years (mean ± SD)		29.7 ± 5.9	**↓27.9** **±** **5.6**	29.3 ± 5.3	29.2 ± 5.0	**↑32.7** **±** **4.5**	31.0 ± 4.9	32.5 ± 4.9
Maternal age *n* (%*N*)	≤25 years	1,940 (26.6%)	**↑593** (**36.9%)**	2,829 (24.5%)	1,697 (22.8%)	**↓69 (4.2%)**	74 (11.8%)	41 (7.5%)
	26–34 years	3,695 (50.8%)	**↓810** (**50.4%)**	6,701 (58.1%)	4,682 (62.8%)	1,036 (63.5%)	**↑409 (65.2%)**	320 (58.7%)
	≥35 years	1,645 (22.6%)	**↓205** (**12.8%)**	1,997 (17.3%)	1,073 (14.4%)	526 (32.3%)	144 (23.0%)	**↑184** (**33.8%)**
BMI at booking (mean ± SD)^a^		**↑30.0** **±** **6.1**	29.4 ± 6.5	29.1 ± 5.7	27.7 ± 5.3	**↓26.6** **±** **4.5**	27.8 ± 5.3	27.2 ± 5.3
BMI at delivery (mean ± SD)^a^		**↑32.2** **±** **5.9**	31.5 ± 6.2	31.7 ± 5.5	30.2 ± 5.1	**↓29.3** **±** **4.4**	30.3 ± 4.9	30.0 ± 5.2
BMI ≥30 kg/m^2^ at delivery^a^		**4,501 (62.2%)**	885 (55.5%)	6,733 (58.8%)	3,517 (47.6%)	**↓634 (39.2%)**	293 (47.7%)	244 (45.3%)
Parity *n* (%*N*)	Nulliparous	**↓1,634 (22.5%)**	448 (27.9%)	3,248 (28.2%)	2,216 (29.7%)	657 (40.3%)	**↑279 (44.5%)**	229 (42.0%)
	Multiparous	4,655 (64.0%)	1,034 (64.3%)	**↑7,777** (**67.5%)**	4,881 (65.5%)	950 (58.3%)	330 (52.6%)	311 (57.1%)
	Grand multi	**↑988 (13.6%)**	126 (7.8%)	502 (4.4%)	355 (4.8%)	24 (1.5%)	18 (2.9%)	**↓5 (0.9%)**
H/o CS in multiparous, *n* (%*N*)		1,767 (31.3%)	**↓300** (**25.9%)**	2,996 (35.8%)	1,666 (31.8%)	305 (31.3%)	106 (30.5%)	**↑122** (**38.6%)**
Assisted conception, *n* (%*N*)		**↑354 (4.9%)**	29 (1.8%)	314 (2.7%)	146 (2.0%)	**↓25 (1.5%)**	11 (1.8%)	26 (4.8%)
Diabetes, *n* (%*N*)		**↑232 (3.2%)**	37 (2.3%)	196 (1.7%)	210 (2.8%)	30 (1.8%)	9 (1.4%)	**↓2 (0.4%)**
Chronic hypertension, *n* (%*N*)		84 (1.2%)	13 (0.8%)	90 (0.8%)	75 (1.0%)	**↑65 (4.0%)**	5 (0.8%)	5 (0.9%)
Thyroid, *n* (%*N*)		751 (10.3%)	99 (6.2%)	710 (6.2%)	**↑891** (**12.0%)**	**↓57 (3.5%)**	27 (4.3%)	40 (7.3%)
Hospital, *n* (%*N*)	Central tertiary	**↑6,638 (91.2%)**	1,316 (81.8%)	7,282 (63.2%)	4,418 (59.3%)	1,028 (63.0%)	389 (62.0%)	**↓236 (43.3%)**
	Periphery	**↓639 (8.8%)**	292 (18.2%)	4,245 (36.8%)	3,034 (40.7%)	603 (37.0%)	238 (38.0%)	**↑309 (56.7%)**
GA at booking *n* (%*N*)	First trimester	2,137 (29.4%)	412 (25.6%)	**↑4,164** (**36.1%)**	2,227 (29.9%)	↓**409 (25.1%)**	158 (25.2%)	162 (29.7%)
	Second trimester	2,484 (34.1%)	↓**481** (**29.9%)**	3,713 (32.2%)	2,634 (35.4%)	**↑598 (36.7%)**	229 (36.5%)	168 (30.8%)

SD, standard deviation; GCC, Gulf Cooperation Council; SE, southeast; BMI, body mass index; CS, cesarean section; GA, gestational age.

Bold values represent the groups with the highest and lowest observations.

a Missing values: BMI at booking: 7.4%, BMI at delivery: 0.74%; arrows represent smallest and largest proportions; *p* < 0.001 for all variables.

Nearly 5% of QG and EA pregnancies were conceived via ART, accounting for the higher rates (2.9%–3.1%) of multiple gestations in these groups (Table 2). The overall incidence of GDM in the cohort was 28.6%, with only the SA group having a higher incidence at 33.8% (Bangladesh highest at 43%). Although only 4.2% of the cohort developed HIP, the incidence was double in SEA and AF (9%), with preeclampsia accounting for >55% of this risk. Anemia was highest in QG and GCC at 18%, compared with SEA (lowest at 4.7%). More than 9% of women in the cohort had PTB, which was highest in SEA and QG (11%–12%). Women in AF had more than three times the risk of postdated birth compared with SEA (16.8% vs. 5.3%, *p* < 0.001). All groups had a similar incidence of COP.

**Table 2:** Risks of antenatal and intrapartum outcomes in the nationality groups, with 95% confidence intervals.

Antenatal/intrapartum outcomes		Qatar *N* = 7,277	GCC *N* = 1,608	Arab *N* = 11,527	South Asia *N* = 7,452	SE Asia *N* = 1,631	Africa *N* = 627	Eur/Ame *N* = 545	*p*-Value
Multiple gestations (%*N*)		**↑**2.9% (2.50, 3.27)	1.6% (1.05–2.29)	2.3% (2.01–2.55)	1.1% (0.91–1.39)	↓**1.0%** (**0.65**–**1.67)**	1.9% (1.09–3.34)	**↑3.1%** (**1.95**–**4.96)**	<0.0001
Anemia (%*N*)		**↑17.8% (16.89–18.65)**	17.5% (15.76–19.47)	9.7% (921–10.30)	10.5% (9.83–11.22)	↓**4.7%** (**3.79–5.86)**	11.3% (9.07–14.05)	6.8% (4.96–9.23)	<0.0001
Cholestasis of pregnancy (%*N*)		1.3% (1.08–1.61)	↓**0.9%** (**0.56–1.54)**	0.9% (0.78–1.13)	**↑1.6%** (**1.37–1.95)**	1.2% (0.79–1.89)	1.1% (0.53–2.32)	1.3% (0.61–2.67)	0.0026
Gestational diabetes (%*N*)		26.0% (24.99–27.04)	25.8% (23.68–28.00)	28.5% (27.66–29.32)	**33.4%** (**32.33–34.50)**	28.6% (26.45–30.87)	23.8% (20.59–27.30)	↓**14.4%** (**11.66–17.57)**	<0.0001
New onset HIP (%*N*)	Total	3.6% (3.22–4.09)	3.3% (2.55–4.32)	↓**3.2%** (**2.93–3.58)**	5.0% (4.57–5.57)	↑**9.4%** (**8.04–10.93)**	↑**9.2%** (**7.13–11.70)**	5.2% (3.60–7.41)	<0.0001
	Gestational hypertension	↓**1.4%** (**1.16–1.70)**	↓**1.4%** (**0.9–2.09)**	1.6% (1.34–1.79)	2.5% (2.14–2.85)	**↑4.1%** (**3.21–5.19)**	**↑3.7%** (**2.47–5.50)**	3.2% (1.97–5.01)	<0.0001
	Preeclampsia	2.2% (1.91–2.59)	1.9% (1.7–2.75)	↓**1.7%** (**1.47–1.94)**	2.6% (2.24–2.96)	**↑5.3%** (**4.29–6.53)**	**↑5.5%** (**3.93–7.55)**	2.0% (3.93–7.55)	<0.0001
Preterm birth (%*N*)		11.3% (10.60–12.06)	9.7% (8.35–11.25)	**↓7.6%** (**7.13–8.10)**	8.8% (8.21–9.50)	**↑11.7%** (**10.19–13.31)**	8.9% (6.94–11.43)	8.8% (6.70–11.50)	<0.0001
Postdate birth (%*N*)		7.6% (7.04–8.26)	8.7% (7.42–10.19)	8.6% (8.07–9.09)	6.2% (5.71–6.81)	**↓5.3%** (**4.29–6.47)**	**↑16.8%** (**14.02–19.88)**	11.0% (8.64–13.92)	<0.0001
Cesarean delivery in singleton (%*N*)		31.4% (30.28–32.44)	**↓24.5%** (**22.45–26.69)**	32.8% (31.91–33.64)	30.6% (29.58–31.68)	37.6% (35.28–40.00)	38.9% (35.09–42.78)	**↑41.3%** (**37.16–45.54)**	<0.0001
Instrumental delivery (%*N*)		**↓7.9% (7.14–8.65)**	9.1% (7.57–10.83)	8.7% (8.07–9.34)	10.0% (9.22–10.87)	**↑14.1%** (**12.13–16.43)**	13.4% (10.32–17.19)	8.0% (5.47–11.59)	<0.0001
PPH (%*N*)		5.9% (5.35–6.43)	**↓5.3%** (**4.29–6.49)**	6.4% (6.00–6.90)	5.7% (5.21–6.27)	**↑12.5%** (**10.99–14.20)**	8.6% (6.66–11.08)	9.4% (7.18–12.11)	<0.0001

GCC, Gulf Cooperation Council; SE, southeast; HIP, hypertension in pregnancy; PPH, postpartum hemorrhage; Instrumental delivery calculated in those who had a vaginal birth; gestational diabetes excludes women with preexisting diabetes; HIP excludes chronic hypertension; arrows represent the smallest and largest proportions; the figures in bold represent clinically relevant differences.

**Figure 3:**
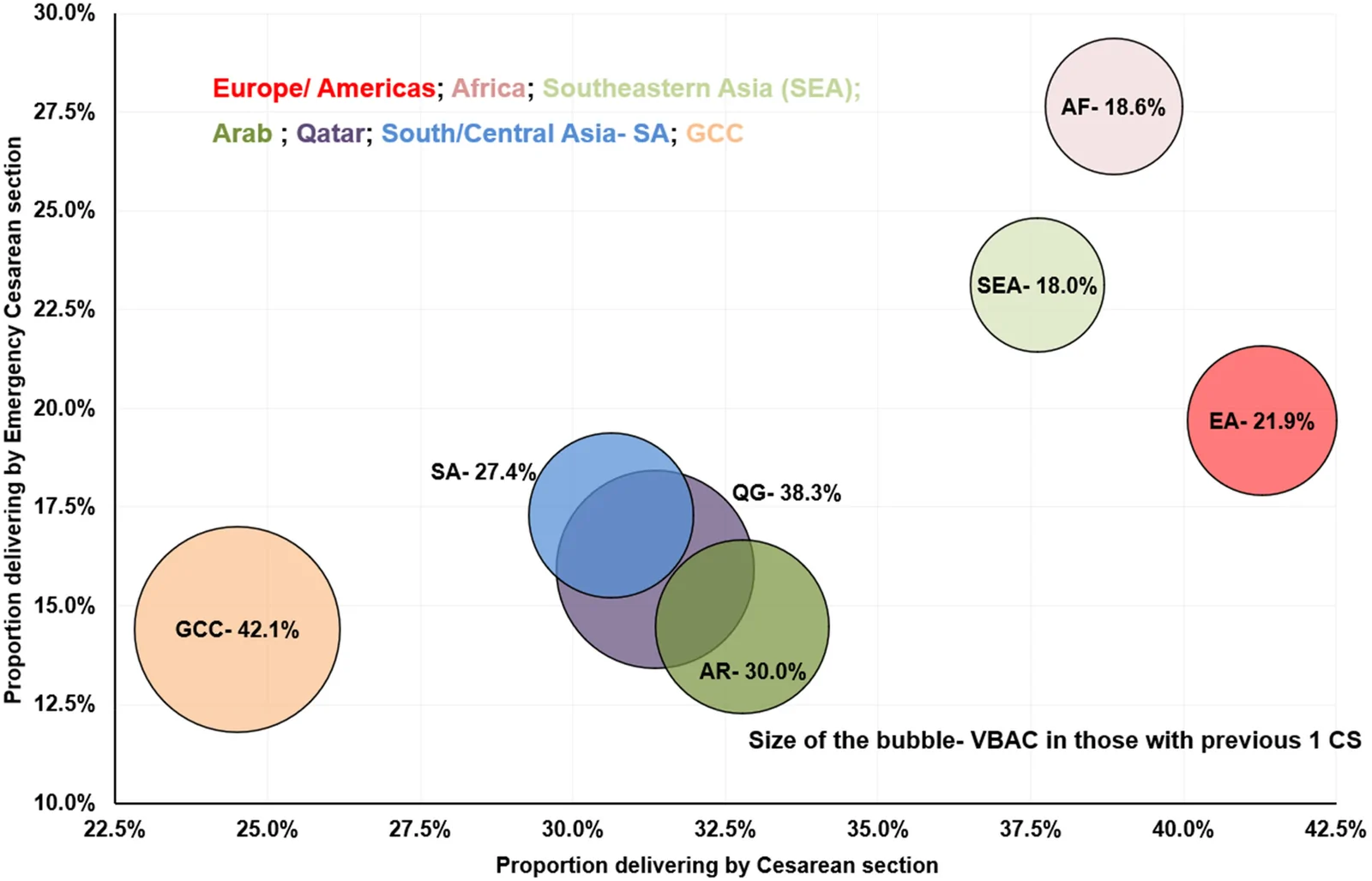
Mode of delivery in singleton births across different nationality groups; number inside bubble represents VBAC: vaginal birth after cesarean in one previous CS. AR, Arab Region; AF, Africa; EA, Europe/Americas; GCC, Gulf Cooperation Council states.

Clinically relevant differences were observed in the mode of delivery in singleton births among the geographic groups (Figure 3). One-third of the women in the cohort delivered by cesarean section, with rates ranging from 24.5% in GCC to 41.3% in EA; half of these were emergency cesareans (16.3% of total). In those with one previous cesarean, GCC and QG had the highest rates of successful VBAC (42.9% and 39.7%, respectively). Although QG, AR, and SA had similar cesarean rates, they differed significantly in VBAC rates, with QG having a high rate compared with SA (27.5%), reflected in the higher rates of emergency cesarean in SA. The lowest VBAC rate was observed in SEA and AF (18%–19%), with AF also having a 27.6% risk of emergency cesarean.

The mean birthweight was lowest in QG, more than 200 g lesser than EA, with nearly four times higher risk of LBW in term babies compared with EA (Table 3). Conversely, 11.4% of the EA group delivered macrosomic babies compared to 3%–4% in the SEA, QG, SA, and GCC groups. Customized birthweight centiles differed significantly between the groups: SEA, QG, and GCC had a median CBW of 39–40 percentile compared with 56.2 percentile in SA. This was reflected in the high risk of SGA in these three groups (16%–17% vs. baseline cohort risk 9.1%) and the higher incidence of LGA infants in SA (17.0% vs. cohort risk 12.7%). Nearly 11% of all term neonates required admission to the NICU, with increased risk in AF and SEA, probably because of increased postdated births in AF and smaller babies in SEA. No clinically or statistically significant differences between groups existed for rare neonatal outcomes.

**Table 3:** Risk of growth and neonatal outcomes in singleton births among the nationality groups, with 95% confidence intervals.

Neonatal outcomes in singleton births	Qatar *N*=7,069	GCC *N*=1,583	Arab *N*=11,266	South Asia N=7,368	SE Asia *N*=1,614	Africa *N*=615	Eur/Ame *N*=528	*p*-Value
Birthweight in g (mean ± SD)	**↓3,132 ± 541**	3,137 ± 547	3,297 ± 519	3,140 ± 534	3,130 ± 554	3,312 ± 591	**↑3,354 ± 594**	<0.0001
LBW in term babies (<2,500 g), *n*(%*N*)	**↑4.5% (3.99–5.01)**	4.2% (3.30–5.39)	2.4% (2.10–2.69)	4.3% (3.87–4.84)	4.0% (3.08–5.12)	1.4% (0.71–2.80)	**↓1.2% (0.55–2.70)**	<0.0001
Macrosomia (>4,000 g), *n*(%*N*)	3.6% (3.20–4.07)	4.1% (3.23–5.20)	6.6% (6.13–7.04)	3.9% (3.49–4.38)	**↓3.3% (2.52–4.27)**	7.8% (5.93–10.21)	**↑11.4% (8.92–14.36)**	<0.0001
Customized birthweight centiles (median, IQR)	↓ 39.2 (16.2–68.0)	**↓ 39.0 (16.2–67.5)**	51.3 (25.4–78.4)	**↑ 56.2 (28.0–82.4)**	40.1 (16.9–68.5)	53.7 (26.7–78.2)	51.6 (24.0–79.5)	0.0001
SGA-CBW <10%, *n*(%*N*)	↑**16.6% (15.72–17.45)**	16.4% (14.68–18.33)	10.4% (9.81–10.93)	9.8% (913–10.49)	↑**16.8% (15.05–18.69)**	**↓8.9% (6.93–11.47)**	10.2% (7.92–13.12)	<0.0001
LGA-CBW >90%, *n*(%*N*)	**↓8.6% (7.93–9.23)**	9.8% (8.42–11.36)	13.4% (12.75–14.01)	**↑17.0% (16.17–17.88)**	8.6% (7.34–10.08)	14.6% (12.06–17.65)	12.7% (10.11–15.81)	<0.0001
NICU in term babies, *n*(%*N*)	**↓9.3% (8.57–9.99)**	9.5% (8.08–11.11)	10.6% (10.01–11.19)	11.4% (10.62–12.13)	13.7% (11.98–15.53)	**↑14.0% (11.34–17.06)**	11.2% (8.72–14.34)	<0.0001
Congenital anomalies (per 1,000 births)	21.5 (18.4–25.2)	18.3 (12.8–26.2)	17.0 (14.7–19.5)	22.9 (19.8–26.6)	18.6 (13.0–16.5)	16.3 (8.8–30.0)	18.9 (10.2–34.8)	0.1145
Chromosomal anomalies (per 1,000 births)	3.0 (1.9–4.6)	3.2 (1.3–7.6)	2.4 (1.6–3.5)	3.3 (2.2–4.9)	2.5 (0.9–6.6)	3.3 (0.8–12.9)	1.9 (0.3–13.3)	0.9499
Stillbirths (per 1,000 births)	4.7 (3.3–6.6)	6.3 (3.4–11.7)	4.8 (3.7–6.3)	6.4 (4.8–8.5)	3.1 (1.3–74)	4.9 (1.6–15.0)	3.8 (0.9–15.0)	0.5707
Neonatal death (per 1,000 births)	3.8 (2.6–5.6)	1.9 (0.6–5.9)	2.9 (2.0–4.0)	3.4 (2.3–5.0)	5.6 (2.9–10.7)	6.5 (2.5–17.3)	5.7 (1.8–17.5)	0.3566

SD, standard deviation; IQR, interquartile range; GCC, Gulf Cooperation Council; SE, Southeast; LBW, low birthweight; SGA, small for gestational age; LGA, large for gestational age; CBW, customized birthweight centiles; NICU, neonatal intensive care unit; arrows represent the smallest and largest proportions; numbers in bold represent clinically relevant differences.

### Adjusted analysis

**Figure 4:**
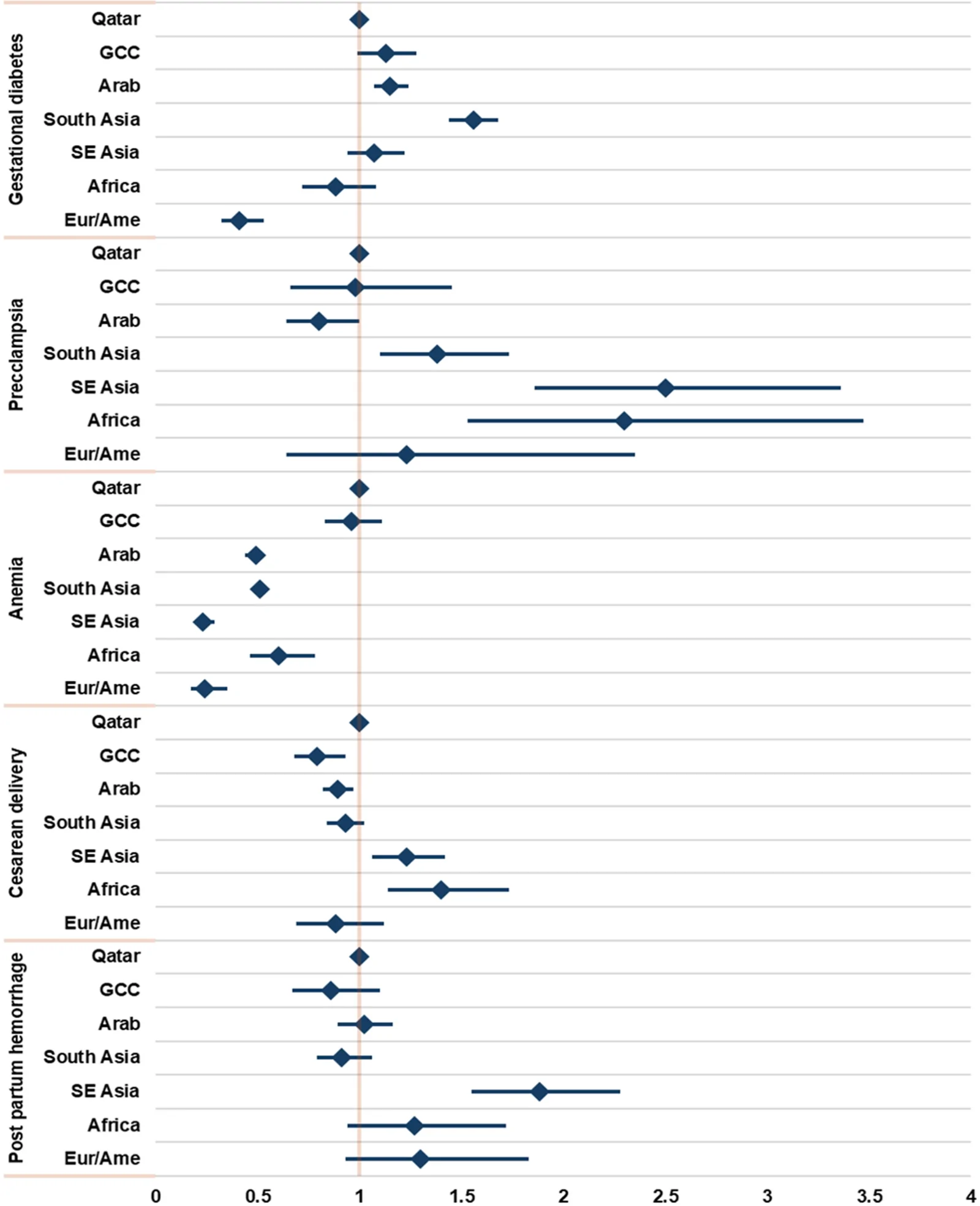
Adjusted odds ratios with 95% confidence intervals for antenatal outcomes; baseline: Qatar; ORs to the right of the reference line represent higher odds of outcome; CIs crossing the reference line are not statistically significant. Adjusted for age, BMI, parity, location of antenatal care, assisted reproduction, and pre-existing comorbidities. Cesarean section adjusted for history of previous cesarean delivery.

**Figure 5:**
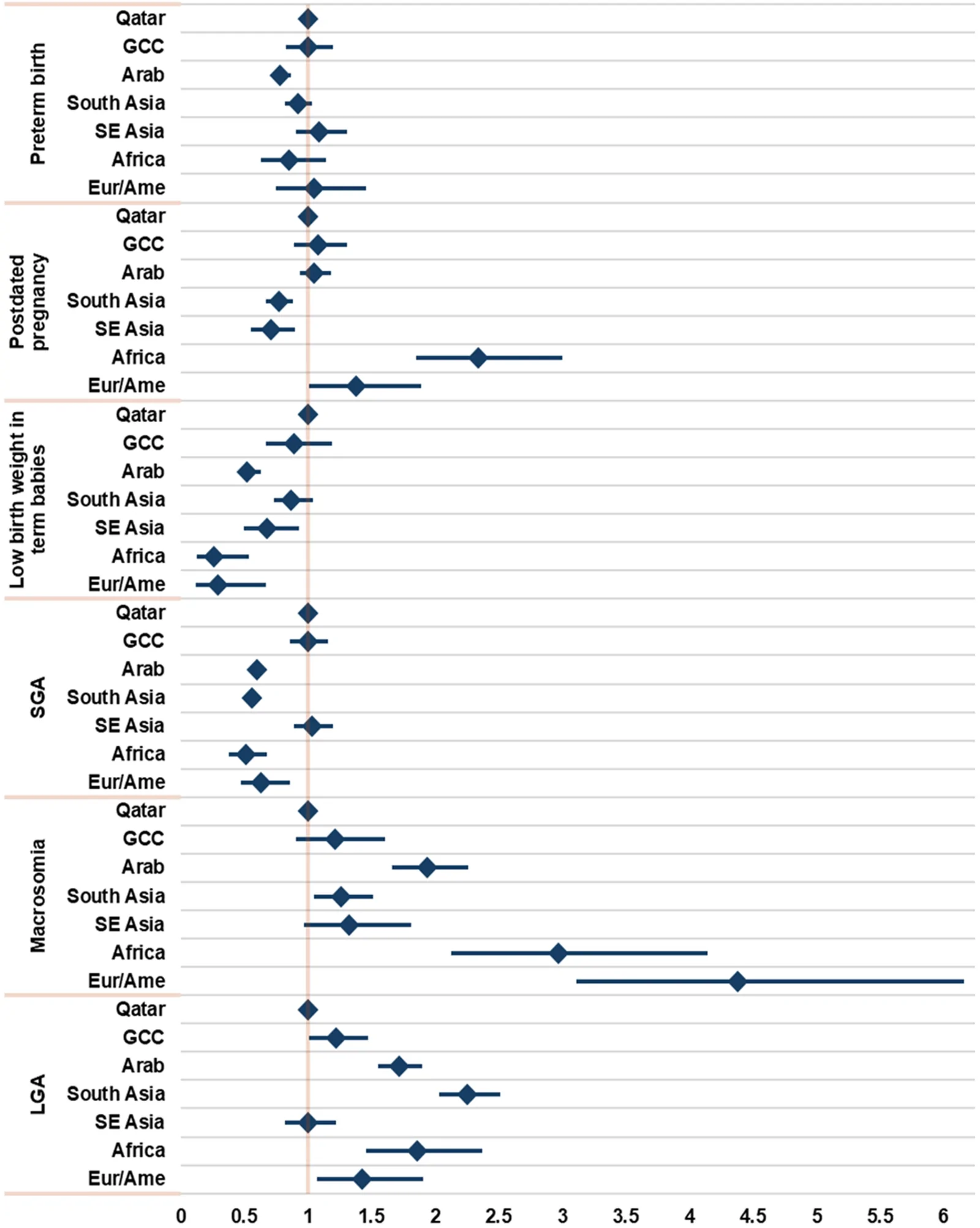
Adjusted odds ratios with 95% confidence intervals for neonatal outcomes; baseline: Qatar; ORs to the right of the reference line represent higher odds of outcome; CIs crossing the reference line are not statistically significant. Adjusted for age, BMI, parity, location of antenatal care, assisted reproduction, and preexisting comorbidities. SGA, small for gestational age; LGA, large for gestational age.

After adjusting for variables listed in Table 1, the AR and SA groups had higher odds of GDM compared with QG (aOR=1.14, CI = 1.06–1.22; and aOR=1.53, CI = 1.42–1.66; *p* < 0.001, respectively), while EA had 51% lower odds (aOR=0.49, CI = 0.38–0.64; *p* < 0.001). The SEA group had 2.5-fold higher odds of preeclampsia (aOR=2.50, CI = 1.86–3.36; *p* < 0.001), while AF had 2.3-fold higher odds (aOR=2.30, CI = 1.53–3.47; *p* < 0.001, respectively). Except for GCC, all groups had significantly lower odds of anemia during pregnancy, with SEA and EA showing 75% lower odds (aOR=0.23, CI = 0.18–0.29 and aOR=0.24, CI = 0.17–0.35; *p* < 0.001, respectively) (Figures 4, 5) (Table 4).

**Table 4:** Antenatal outcomes in the nationality groups, adjusted odds ratios and 95% confidence intervals.

Antenatal outcomes		Qatar *N* = 7,277	GCC *N* = 1,608	Arab *N* = 11,527	South Asia *N* = 7,452	SE Asia *N* = 1,631	Africa *N* = 627	Eur/Ame *N* = 545
Anemia *N* = 30,441	aOR (95% CI)	1	0.96 (0.83–1.11)	0.49 (0.44–0.53)	0.51 (0.46–0.56)	0.23 (0.18–0.29)	0.60 (0.46–0.78)	0.24 (0.17–0.35)
	*p*-Value		0.6198	<0.0001	<0.0001	<0.0001	0.0001	<0.0001
Gestational diabetes *N* = 29,726	aOR (95% CI)	1	1.13 (0.99–1.28)	1.15 (1.07–1.24)	1.56 (1.44–1.68)	1.07 (0.94–1.22)	0.88 (0.72–1.08)	0.41 (0.32–0.53)
	*p*-Value		0.0709	0.0001	<0.0001	0.2927	0.2166	<0.0001
Preeclampsia *N* = 30,104	aOR (95% CI)	1	0.98 (0.66–1.45)	0.80 (0.64–1.00)	1.38 (1.10–1.73)	2.50 (1.86–3.36)	2.30 (1.53–3.47)	1.23 (0.64–2.35)
	*p-*V*alue*		0.9134	0.0533	0.0049	<0.0001	0.0001	0.5361
Preterm birth *N* = 30,440	aOR (95% CI)	1	1.00 (0.83–1.20)	0.78 (0.70–0.87)	0.92 (0.82–1.03)	1.09 (0.91–1.31)	0.85 (0.63–1.14)	1.05 (0.75–1.46)
	*p*-Value		0.9893	<0.0001	0.1588	0.3407	0.2776	0.7885
Postdate birth *N* = 30,440	aOR (95% CI)	1	1.08 (0.89–1.31)	1.05 (0.94–1.18)	0.77 (0.67–0.88)	0.71 (0.55–0.90)	2.34 (1.85–2.97)	1.38 (1.01–1.89)
	*p*-Value		0.4516	0.3777	0.0001	0.0052	<0.0001	0.0412
Cesarean delivery in singleton *N* = 29,823	aOR (95% CI)	1	0.79 (0.68–0.93)	0.89 (0.82–0.97)	0.93 (0.84–1.02)	1.23 (1.06–1.42)	1.40 (1.14–1.73)	0.88 (0.69–1.12)
	*p*-Value		0.0050	0.0089	0.1175	0.0060	0.0015	0.2938
PPH, *n*(%*N*) *N* = 30,441	aOR (95% CI)	1	0.86 (0.67–1.10)	1.02 (0.89–1.16)	0.91 (0.79–1.06)	1.88 (1.56–2.28)	1.27 (0.94–1.72)	1.30 (0.93–1.83)
	*p*-Value		0.2261	0.8213	0.2177	<0.0001	0.1247	0.1281

GCC, Gulf Cooperation Council; SE, Southeast; HIP, hypertension in pregnancy; PPH, postpartum hemorrhage; aOR, adjusted odds ratios; CI, confidence intervals, adjusted for maternal age, parity, BMI, location of delivery, and preexisting comorbidities; gestational diabetes excludes women with preexisting diabetes.

The odds of preterm birth were similar across groups, except for AR, with 22% lower odds (aOR=0.78, CI = 0.70–0.87, *p* < 0.001). Postdated pregnancies were 2.34 and 1.38 times higher in AF (CI = 1.85–3.0) and EA (CI = 1.01–1.89), respectively. The SEA group had nearly twice the odds of PPH (aOR=1.88, CI = 1.55–2.28, *p* < 0.001). NICU admission in term babies was similar across groups, except for AR, with a 16% reduction in odds (aOR=0.84, CI = 0.75–0.94, *p* < 0.001). Fetal growth varied between the groups, with AR, SA, AF, and EA having statistically significantly reduced odds of SGA. SEA and QG had similar odds of LGA babies, while all other groups had significantly higher odds, with SA showing the highest odds, with aOR=2.25 (CI = 2.03–2.51, *p* < 0.001), followed by AF (aOR=1.86, CI = 1.46–2.37, *p* < 0.001) and AR (aOR=1.72, CI = 1.55–1.90, *p* < 0.001). While GCC, AR, and SA had lower odds of cesarean, AF and SEA had higher odds (aOR=1.40, CI = 1.14–1.73; *p* = 0.001 and aOR=1.23, CI = 1.06–1.42; *p* = 0.006, respectively).

## Discussion

This study demonstrates a variation in perinatal outcomes across ethnogeographic groups. After adjusting for prepregnancy factors and using Qataris as the reference, SA had the highest odds of GDM and LGA; SEA had the lowest odds of anemia and VBAC, but the highest odds of HIP and PPH; AF had the highest odds of postdated birth; GCC had highest rate of VBAC and lowest rates of cesarean; Arab women had the lowest odds of PTB and admission to NICU; and EA had lowest odds of GDM, but highest rates of macrosomia, multiple gestations, and cesarean delivery.

Ethnicity—linked to a person's cultural beliefs and customs (5, 11, 12)—has become more relevant to health outcomes in recent generations, particularly in populations with mixed-race individuals. Ethnicity is also closely associated with the geographical location of one's birth, upbringing, and/or citizenship (nationality). In obstetrics, this relevance arises from differences in factors impacting pregnancy outcomes such as maternal age at conception, parity, consanguinity, diet, lifestyle, and healthcare choices. Qatar’s maternity population is predominantly expatriate, with women from over 100 countries. For these women, additional factors such as language barriers, availability of social support, and adaptation to new environmental conditions, directly linked to their nationalities, become relevant to maternal outcomes. Qatar’s public healthcare system is freely and uniformly accessible to all residents. Large meta-analyses suggest that adverse perinatal outcomes in underserved ethnic groups in the West are due to disparities in healthcare access and differences in treatment (2). Therefore, the multinational sample in this middle- to high-income society in our study is uniquely poised to evaluate the impact of nationality, potentially with reduced confounding by factors such as healthcare access or income.

The outcome most widely studied in association with nationality is GDM. International guidelines recommend early screening for women with a higher background (ethnic) risk for diabetes such as SA (13), due to differences in attitudes toward screening, shorter interpregnancy intervals, and poorer glycemic control in between pregnancies. Large studies from multinational settings, including Australia, have reported higher GDM rates in SA and SEA, after adjusting for age, BMI, and parity (14, 15). Qatar employs universal screening for GDM based on World Health Organization (WHO) criteria (16). In our study, SA women—particularly those from Bangladesh, Nepal, and India—had the highest incidence of GDM, with nearly 50% higher odds than most groups, due to the underlying genetic susceptibility (17) rather than any socioeconomic factor. Similar findings were reported in a New Jersey study, where SA women had nearly four times higher incidence compared with White women (18). Overall, the GDM prevalence in our study was double that of global estimates (19), likely since nearly 50% of women belonged to Qatar, Egypt, and India (GDM rate of 26% vs. 32% vs. 35%, respectively), justifying the universal screening currently practiced. The GDM rates may also be explained by higher obesity compared with rates in their home countries, likely because of expatriate lifestyles.

We report a risk of preeclampsia ranging from 1.7% to 5.4%, with the highest incidence in SEA and AF. Systematic reviews have reported a higher prevalence in the Philippines (among the Eastern countries) and low-income African countries (20). The global differences can be attributed to socioeconomic and healthcare disparity, with the low-income countries particularly disadvantaged. The overall risk of preeclampsia in our setup was only 2.3%; however, it 's still significantly higher in women from SEA and Africa after adjusting for confounders, suggesting genetic or dietary etiologies. Interestingly, BMI was not a factor here, as SEA has the lowest mean BMI at delivery. The higher preexisting hypertension risk in this group hints at genetic susceptibility. The Philippines have reported higher maternal mortality (nearly 37%) due to HIP, with studies identifying preeclampsia-specific genes in pregnant women of this nation (21). Studies from USA report a higher risk in women of African origin, attributed to poorer SES, access to healthcare, and associated chronic stress (22). Contrary to this belief, a large Californian birth cohort study demonstrated that improvement in SES did not change preeclampsia risk in these women (23), as evident in our study.

Similarly, a doubling of PTB has been reported among women of African origin living in the Western world, while other ethnicities did not differ (24). The higher risk of iatrogenic and spontaneous PTB in this group has been attributed to their chronic detrimental socioeconomic circumstances and differences in estimation of GAs, rather than due to any specific genetic susceptibility (25). This difference was not observed in our cohort, with AF and EA women having the same risk. The differences in aORs were not clinically or statistically significant when compared with QG, reflecting the universal, equitable, and readily accessible emergency healthcare services in Qatar.

Contrarily, AF women had a high rate of postdated births, reflecting the high nulliparity and overall lesser complicated pregnancies in this group. Studies have highlighted the much higher risk of emergency cesarean in women induced for postdated pregnancies (26); this was evident from the high rate of emergency cesarean in AF women in our cohort. Similarly, significantly higher odds of cesarean in SEA women reflect the higher prevalence of preexisting hypertension and new-onset HIP. Among AR women, 45% of women from Egypt underwent cesarean since two-fifths had undergone a previous cesarean. The preference for a repeat cesarean is evident in the high cesarean rate in their country (>60%) (27). In contrast, 36% of Indian women in our cohort underwent cesarean. This is much higher than the national rate of 21.5% in India, although studies show that rates can increase to 30%–40% in educated urban mothers (28), which explains our findings. Women in QG and GCC had high VBAC rates due to the cultural expectations of larger families, compared with SA and SEA, where 1–2 offspring is the norm.

Anemia in pregnancy is concerningly higher in QG and GCC. This may be due to higher bariatric surgery-associated malnourishment in the nationals, as reported previously (29). Dietary habits, obesity, and menstrual irregularities, including menorrhagia, often result in young Qatari girls entering their reproductive age with chronic anemia. In addition, inherited anemias have tremendously increased in Qatar over the past decade (30). Interestingly, SA and AF regions are known to have high rates of iron deficiency and inherited anemias during pregnancy (31). In our cohort, expatriates from these regions fared much better than nationals, with markedly reduced risk compared with reports from their countries of birth.

PPH remains a global concern, mainly in middle- to low-income countries, accounting for significant mortality (32). Our cohort had an overall rate of 6.5% which doubled in SEA (particularly among women from the Philippines and Indonesia). Overall, four in 1,000 women had >2,000 mL blood loss, which increased to 10 in 1,000 SEA women. The higher cesarean rate does not account for this, as even among SEA women delivering vaginally, 13.5% had PPH. In a clinical setup where medications, surgical interventions, and expertise are available to combat PPH, it 's difficult to explain our finding if not attributing it to the inherent susceptibility in this nationality group. The highest rates of maternal death due to PPH are reported from the African continent (33); however, in our cohort, AF was not at an increased risk, explained by the availability of resources.

Neonatal complications were comparable among groups despite differences in maternal complications. QG, GCC, and SEA women had smaller babies; in SEA, this could be due to higher preeclampsia risk and associated growth restriction. The higher NICU admission in this group also highlights possible placental pathology. However, in QG and GCC, the smaller babies did not increase NICU admission, hinting at constitutional variations rather than pathological growth restriction. The AF group had a higher NICU admission, possibly due to postdated pregnancies and cesarean delivery; these differences were not clinically relevant in adjusted analyses.

### Clinical relevance

Certain outcomes were strikingly different among certain nationality groups. Nationality or ethnic origin, therefore, is an essential element of appropriate antenatal risk assessment and needs to be incorporated into local and international guidelines. Women from SA who are at a higher risk of developing GDD and type 2 diabetes in the future should be advised to undergo an earlier GTT by 16 weeks and advised to evaluate their glucose tolerance 6 weeks postpartum. This study provides interesting insights into the indications for cesarean in the country. Identifying nationality groups with higher rates of repeat cesarean can help direct the focus of various interventions aimed at reducing the cesarean rate.

Further research exploring the use of non-invasive prenatal testing and uterine artery Doppler in predicting the development of preeclampsia in high-risk groups such as SEA and AF is warranted. Exploration of prepregnancy anemia profiles in adolescent girls from Qatar and GCC countries is also warranted to evaluate the prevalence and causes. Steps can be taken to improve the prepregnancy health of these girls, such as regulating menstrual cycles, iron supplementation, and improving BMI.

### Strengths and limitations

This study is the largest from the region to explore perinatal outcomes among women across different ethnogeographic groups, using high-quality data from the pre-COVID period. The findings are relevant to our clinical practice, as the nationality of pregnant women is overlooked during risk assessment. Women belonging to high-risk nationality groups should be identified and directed along appropriate antenatal care pathways, thereby reducing maternal morbidity. Exposure classification was based on internationally accepted ethnogeographic grouping rather than race. Countries such as Egypt, Sudan, and Somalia were included in the Arab group due to shared language, religion, and culture. The EA group had a heterogeneous mix of women from Europe, Australia, and the Americas due to fewer numbers; most were Caucasian and culturally distinct from the other groups, allowing meaningful comparisons. However, the heterogeneity in this group must be considered when interpreting the results pertaining to this group. Further studies, with larger numbers permitting more homogeneous grouping in EA, are warranted.

Despite adjusted analyses, residual confounding remains a possibility. For example, we have not included any socioeconomic variables such as occupation, income, or education in the analysis. However, government healthcare services are equitable and accessible regardless of SES or insurance coverage. It 's less likely that these variables would have affected maternal outcomes in our setup. In addition, the expatriate community mostly consists of young migrants and their spouses with satisfactory prepregnancy health. Nevertheless, the unmeasured confounding effect of SES is real and can potentially bias the results; hence, the findings from this study should be interpreted with caution. We have not included pregnancies ending before 24 weeks’ gestation, potentially underestimating outcomes such as anomalies; however, this is unlikely to vary according to nationality. Finally, there was a preference among the expatriate community to deliver at peripheral hospitals; however, all hospitals fall under the same medical corporation, following common clinical guidelines, and this is unlikely to bias the estimation of outcomes.

## Conclusion

This study demonstrates associations between perinatal outcomes and maternal nationality. Major maternal complications differed statistically significantly between the groups, independent of other maternal variables. Incorporating nationality in risk assessment scores is valid and essential for accurate risk management. The disparities in outcomes observed in this study could potentially be due to inherent genetic constitutions, dietary differences, cultural and traditional lifestyle, and healthcare choices. As in other branches of medicine, the role of ancestry and genetic susceptibility is relevant for risk assessment in obstetrics. Understanding these differences is essential for adequate risk assessment, and further genetic and anthropological studies are warranted to confirm these findings.

## Data Availability

The data analyzed in this study are subject to the following licenses/restrictions: Data used for this study are restricted due to institutional confidentiality policies. However, anonymized data can be made available by the corresponding author upon reasonable request. Requests to access these datasets should be directed to Fathima Minisha, fminisha@hamad.qa.

## References

[1] Race and genetics versus ‘race’ in genetics: A systematic review of the use of African ancestry in genetic studies—PMC [Internet]. Available online at: https://pmc.ncbi.nlm.nih.gov/articles/PMC8604262/ (accessed December 28, 2024). 10.1093/emph/eoab018 PMC8604262 34815885

[2] Sheikh J Allotey J Kew T Fernández-Félix BM Zamora J Khalil A. Effects of race and ethnicity on perinatal outcomes in high-income and upper-middle-income countries: an individual participant data meta-analysis of 2 198 655 pregnancies. Lancet. (2022) 400(10368):2049–62. 10.1016/S0140-6736(22)01191-6 36502843

[3] Grobman WA Parker CB Willinger M Wing DA Silver RM Wapner RJ. Racial disparities in adverse pregnancy outcomes and psychosocial stress. Obstet Gynecol. (2018) 131(2):328–35. 10.1097/AOG.0000000000002441 29324613 PMC5785441

[4] Adverse pregnancy outcomes attributable to socioeconomic and ethnic inequalities in England: a national cohort study—PubMed [Internet]. Available online at: https://pubmed.ncbi.nlm.nih.gov/34735797/ (accessed December 28, 2024). 10.1016/S0140-6736(21)01595-6 34735797

[5] Flanagin A Frey T Christiansen SL, AMA Manual of Style Committee. Updated guidance on the reporting of race and ethnicity in medical and science journals. JAMA. 2021;326(7):621–7. 10.1001/jama.2021.13304 34402850

[6] Vandenbroucke JP Von Elm E Altman DG Gøtzsche PC Mulrow CD Pocock SJ. Strengthening the Reporting of Observational Studies in Epidemiology (STROBE): explanation and elaboration. PLoS Med. (2007) 4(10):e297. 10.1371/journal.pmed.0040297 17941715 PMC2020496

[7] Abdul-Rasoul MM. Obesity in children and adolescents in Gulf countries: facts and solutions. Av En Diabetol. (2012) 28(3):64–9. 10.1016/j.avdiab.2012.04.001

[8] League of Arab States (LAS) and the EU | EEAS [Internet]. Available online at: https://www.eeas.europa.eu/eeas/league-arab-states-las-and-eu_en (Accessed November 25, 2024).

[9] United Nations Statistics Division. United Nations Statistics Division—Methodology—Standard Country or Area Code [Internet]. Available online at: https://unstats.un.org/unsd/methodology/m49/ (accessed November 28, 2024).

[10] StataCorp. Stata Statistical Software: Release 18. College Station, TX: StataCorp LLC (2023).

[11] Egede LE. Race, ethnicity, culture, and disparities in health care. J Gen Intern Med. (2006) 21(6):667–9. 10.1111/j.1525-1497.2006.0512.x 16808759 PMC1924616

[12] Yuen L Wong VW Simmons D. Ethnic disparities in gestational diabetes. Curr Diab Rep. (2018) 18(9):68. 10.1007/s11892-018-1040-2 30039471

[13] Evidence | Diabetes in Pregnancy: Management from Preconception to the Postnatal Period | Guidance | NICE [Internet]. London: NICE (2015). Available online at: https://www.nice.org.uk/guidance/ng3/evidence (accessed December 14, 2024).

[14] McDonald R Karahalios A Le T Said J. A retrospective analysis of the relationship between ethnicity, body mass Index, and the diagnosis of gestational diabetes in women attending an Australian antenatal clinic. Int J Endocrinol. (2015) 2015:297420–7. 10.1155/2015/297420 26504462 PMC4609521

[15] Wong VW Lin A Russell H. Adopting the new world health organization diagnostic criteria for gestational diabetes: how the prevalence changes in a high-risk region in Australia. Diabetes Res Clin Pract. (2017) 129:148–53. 10.1016/j.diabres.2017.04.018 28528075

[16] Diagnostic Criteria and Classification of Hyperglycaemia First Detected in Pregnancy [Internet]. Geneva: World Health Organization (2013). (WHO Guidelines Approved by the Guidelines Review Committee). Available online at: http://www.ncbi.nlm.nih.gov/books/NBK169024/ (accessed December 14, 2024). 24199271

[17] Rani PR Begum J. Screening and diagnosis of gestational diabetes mellitus, where do we stand. J Clin Diagn Res JCDR. (2016) 10(4):QE01–4. 10.7860/JCDR/2016/17588.7689 27190902 PMC4866200

[18] Sanchalika A Teresa J. Risk of gestational diabetes among south Asian immigrants living in New Jersey—a retrospective data review. J Racial Ethn Health Disparities. (2015) 2(4):510–6. 10.1007/s40615-015-0099-6 26863557

[19] Eades CE Burrows KA Andreeva R Stansfield DR Evans JMM. Prevalence of gestational diabetes in the United States and Canada: a systematic review and meta-analysis. BMC Pregnancy Childbirth. (2024) 24(1):204. 10.1186/s12884-024-06378-2 38491497 PMC10941381

[20] Zhang N Tan J Yang H Khalil RA. Comparative risks and predictors of preeclamptic pregnancy in the Eastern, Western and developing world. Biochem Pharmacol. (2020) 182:114247. 10.1016/j.bcp.2020.114247 32986983 PMC7686229

[21] Amosco MD Tavera GR Villar VAM Naniong JMA David-Bustamante LMG Williams SM. Non-additive effects of ACVR2A in preeclampsia in a Philippine population. BMC Pregnancy Childbirth. (2019) 19(1):11. 10.1186/s12884-018-2152-z 30621627 PMC6323705

[22] Adler NE Rehkopf DH. U.S. sisparities in health: descriptions, causes, and mechanisms. Annu Rev Public Health. (2008) 29:235–52. 10.1146/annurev.publhealth.29.020907.090852 18031225

[23] Ross KM Dunkel Schetter C McLemore MR Chambers BD Paynter RA Baer R. Socioeconomic status, preeclampsia risk and gestational length in black and white women. J Racial Ethn Health Disparities. (2019) 6(6):1182–91. 10.1007/s40615-019-00619-3 31368002

[24] Schaaf JM Liem SMS Mol BWJ Abu-Hanna A Ravelli ACJ. Ethnic and racial disparities in the risk of preterm birth: a systematic review and meta-analysis. Am J Perinatol. (2013) 30(6):433–50. 10.1055/s-0032-1326988 23059494

[25] Variations in gestational length and preterm delivery by race, ethnicity and migration—ScienceDirect [Internet]. Available online at: https://www-sciencedirect-com.ez.lshtm.ac.uk/science/article/pii/S1521693415001613 (accessed December 22, 2024). 10.1016/j.bpobgyn.2015.08.017 26458997

[26] Tarimo CS Mahande MJ Obure J. Prevalence and risk factors for caesarean delivery following labor induction at a tertiary hospital in north Tanzania: a retrospective cohort study (2000–2015). BMC Pregnancy Childbirth. (2020) 20:173. 10.1186/s12884-020-02861-8 32188409 PMC7079438

[27] Al Rifai RH. Trend of caesarean deliveries in Egypt and its associated factors: evidence from national surveys, 2005–2014. BMC Pregnancy Childbirth. (2017) 17(1):417. 10.1186/s12884-017-1591-2 29237410 PMC5729511

[28] Neethi Mohan V Shirisha P Vaidyanathan G Muraleedharan VR. Variations in the prevalence of caesarean section deliveries in India between 2016 and 2021—an analysis of Tamil Nadu and Chhattisgarh. BMC Pregnancy Childbirth. (2023) 23(1):622. 10.1186/s12884-023-05928-4 37649006 PMC10466745

[29] Al-Dewik NI Samara M Mahmah A Al-Dewik A Abou Nahia S Abukhadijah HJ. Maternal and neonatal risks and outcomes after bariatric surgery: a comparative population based study across BMI categories in Qatar. Sci Rep. (2024) 14(1):27107. 10.1038/s41598-024-69845-y 39511221 PMC11543688

[30] Tang H Zhang N Liu X Xiao H Zhang H Zhou K. Incidence trends of inherited anemias at the global, regional, and national levels over three decades. J Epidemiol Glob Health. (2024) 14(1):72–85. 10.1007/s44197-023-00170-9 38079097 PMC11043255

[31] Goodarzi E Beiranvand R Naemi H Darvishi I Khazaei Z. Prevalence of iron deficiency anemia in Asian female population and human development index (HDI): an ecological study. Obstet Gynecol Sci. (2020) 63(4):497–505. 10.5468/ogs.19196 32689776 PMC7393760

[32] World Health Organization. A roadmap to combat postpartum haemorrhage between 2023 and 2030 [Internet]. Available online at: https://www.who.int/publications/i/item/9789240081802 (accessed December 25, 2024).

[33] Forbes G Akter S Miller S Galadanci H Qureshi Z Fawcus S. Factors influencing postpartum haemorrhage detection and management and the implementation of a new postpartum haemorrhage care bundle (E-MOTIVE) in Kenya, Nigeria, and South Africa. Implement Sci. (2023) 18(1):1. 10.1186/s13012-022-01253-0 36631821 PMC9832403

